# Violation of ethical principles in clinical research. Influences and possible solutions for Latin America

**DOI:** 10.1186/1472-6939-13-35

**Published:** 2012-12-16

**Authors:** Borys Alberto Cornejo Moreno, Gress Marissell Gómez Arteaga

**Affiliations:** 1 Research Coordinator Medical Associate, Planning and Institutional Liaison Coordination, Healthcare Services Management, The Mexican Institute of Social Security, Hidalgo Delegation, Delegación Hidalgo, Boulevard Luis Donaldo Colosio n° 516, Pachuca de Soto, Hidalgo, CP 42070, Mexico; 2 Planning and Institutional Liaison Coordinator, Healthcare Services Management, The Mexican Institute of Social Security, Hidalgo Delegation, Boulevard Luis Donaldo Colosio n° 516, Canutillo, Pachuca de Soto, Hidalgo, CP 42070, Mexico

**Keywords:** Informed consent, Autonomy, Genomics, Ethical, Justice, Nonmaleficence, Beneficence

## Abstract

**Background:**

Even though we are now well into the 21st century and notwithstanding all the abuse to individuals involved in clinical studies that has been documented throughout History, fundamental ethical principles continue to be violated in one way or another.

**Discussion:**

Here are some of the main factors that contribute to the abuse of subjects participating in clinical trials: paternalism, improper use of informed consent, lack of strict ethical supervision, pressure exerted by health institutions to increase the production of scientific material, and the absence of legislation regarding ethics in terms of health care and research. Are researchers ready to respect fundamental ethical principles in light of the ample window of information provided by individual genomes, while defending the rights of the subjects participating in clinical studies as a major priority?

**Summary:**

As one of the possible solutions to this problem, education regarding fundamental ethical principles is suggested for participants in research studies as an initial method of cognitive training in ethics, together with the promotion of ethical behavior in order to encourage the adoption of reasonable policies in the field of values, attitudes and behavior.

## Background

Ethical principles such as autonomy, beneficence, nonmaleficence and justice [1] are useful for both researchers and participants in a clinical trial as an overarching frame of reference. They are also helpful for making decisions concerning each individual case, as a means of avoiding abusive situations in experimental processes that involve human beings. Nevertheless, close supervision of both researchers and research subjects is necessary to achieve compliance with ethical principles.

Unfortunately, some of the populations that are most exposed to abuse are vulnerable groups (i.e. confined or chronically ill individuals, elderly patients, pregnant women, single mothers, economically disadvantaged populations), whose main weakness is a latent need to be treated, receive medical care or be assisted in some other way. These people are an easy target for abuses related to their own rights, as their situation forces them to look for help without having many options and generally being less informed about their rights. Some questions arise when considering this situation in a generation that should be familiar with the ethical principles of scientific investigation: Are we ready to act ethically in the genomics era, when a significant number of illnesses and their prognoses are directly related to genome analysis? Would it be a better option to make the principles known in order to empower vulnerable populations, helping them to be aware of their rights and educating them on the ways in which they could suffer abuse? Would this prevent manipulation and promote a change of attitude in terms of values, such as respect and tolerance, in our generation?

## Discussion

### Factors that promote noncompliance with ethical principles

#### Lack of strict ethical surveillance

There is currently no evidence of strict ethical surveillance in many countries of Latin America that participate in clinical investigation, thus making the abuse of ethical principles very likely, as well as factual. The lack of strict ethical supervision encourages the researcher not to inform the participants of their rights. This enables the researcher to have more freedom for the development of the trial. Although it might be assumed that all the necessary information concerning individual rights will be provided by him, in fact this rarely occurs.

### Paternalism

An additional factor is paternalism, a situation that allows the treating physician or investigator to perform actions without consulting the patient, and in some cases even with a certain level of concealment that allows them, according to their own judgment, to act in the most convenient manner [2]. In this setting, the patients see the physician/researcher as their savior, voluntarily giving up their ability to make decisions and subtly losing their autonomy throughout the process. A frequent mindset in participant subjects that are simultaneously being treated in clinical trials is to think of the physician as the person responsible for deciding the treatment, since they have the necessary training and a better capability for making illness-related decisions. This blind trust in the physician/researcher allows him to act almost without limits, minimizing what patients might understand about their illness or what their genetic information might represent. The only thing needed from the patients from this moment on will be their signatures on a document that they rarely understand but acknowledge as a requirement for being treated [3], the informed consent form.

### Informed consent (IC)

The IC form was created to protect the rights of research subjects. However, it currently appears to be used more as way for investigators to protect themselves from any legal situation in which they could be indicted for suspicion of abuse. It has been done as a routine procedure without regard to its real meaning. There are currently very few studies analyzing the effectiveness of the IC form^3^ in Latin America or verifying whether the advantages and disadvantages of a trial are fully explained to the patient –i.e. whether or not there are benefits or if these benefits will be direct or long-term-. These kinds of circumstances are frequently covered or not explicitly explained in the IC form, due to the repercussions they might have on recruitment. In an effort to avoid unnecessary difficulties in the research process, only positive expectations are presented, such as: "this study will help discover the cure for your illness", without a clarifying statement to explaining that the probability of success could be extremely low.

The Informed Consent form was created to help the patients understand all the information related to every aspect of the study they would participate in and −particularly important in our times− full disclosure by the investigator of every aspect concerning the experiment, including risks, benefits and interests; as well as complete comprehension of the information by the patient. Where the research involves studies in genetics, it is essential to add the assurance of confidentiality for the obtained information, since this provides substantial and valuable data for both treatment and prognosis [4].

The concealment of information or the omission of any part of this document also constitutes a form of abuse, even though it may well be an everyday practice in some Latin American countries. When there is no interest in verifying the level of understanding of the patients concerning the study in which they participate, autonomy and justice are voluntarily left out of the equation and the principle of “nonmaleficence " is at risk. Autonomy is also threatened when, not being aware of all the aspects of a particular situation, patients are asked to make decisions that make them responsible for the direct consequences of an action. When only a partial truth is revealed, other people make the actual decisions and autonomy becomes a mere simulation. Justice is placed at risk because the subjects do not receive what they are entitled to when participating in a study, which is to know the reality concerning their situation, the process and the information currently available in the field regarding their entity. The principle of nonmaleficence is also endangered when there is a risk of damaging the subjects while deciding on their behalf, since we do not know if our decision and the decision of the participant are equal.

### Lack of healthcare legislation to promote the protection of vulnerable subjects

The General Healthcare Law of Mexico, as updated on June 7, 2012 does explicitly mention the risk of incurring these circumstances. The lack of attention to the aforementioned factors in different countries contributes to an inadequate prevention of abuse [5].

### Other related factors

There is pressure exerted by healthcare institutions on investigators in terms of the productivity of scientific material. Since a large portion of the research funds in both developed and developing countries come from government budgets and from non-governmental organizations, productivity goals are always considered. This is one of the reasons why scientists are pressured to produce; and to do so, they are tempted to abridge the information given to subjects, leaving out what they consider irrelevant for the knowledge of patients, thus accelerating the process of recruitment and production. In cases where transparency complicates the research process, the truth could not be prioritized, which in turn limits value-driven actions.

Are these kinds of acts justifiable?

Notwithstanding the fact that everyone is accountable for their actions, responsibility mostly lies with decision makers who know the consequences of previously noticed actions. Regardless of the motivation, this is not justifiable to the point of leaving ethical principles aside and adopting purely utilitarian stances where "the means are justified by the end," without regard to beneficence, justice and autonomy of the subjects. This is analogous to what happened in the past when experiments were performed on human beings without any consent or benefits and by infringing on their rights.

Article 4 of the Political Constitution of Mexico, in the first item referring to personal rights, states that: "every person has the right to health care protection" [6] in addition to stating the right to social security and physical and mental healthcare, as second-generation human rights [7]. When omissions of information happen, healthcare is not protected, nor is social security provided.

### Possible solutions

Ethics committees, established for the supervision of international treaties such as the Declaration of Helsinki [8], could be overhauled in terms of surveillance measures to allow a stricter monitoring of patients participating in clinical trials. For instance, accessibility to ethics committees could be useful for subjects in trials, who would be interviewed and asked to answer questionnaires adapted to their academic training; aiming to explore their level of understanding about the study they are involved in and to assess their actual perception. Despite the fact that in recent years the level of comprehension of participants in clinical trials has been a controversial subject, it is necessary to acknowledge that anyone who voluntarily participates in a research study and signs an IC form deserves, and has a right to an explanation according to their own cognitive capabilities. Likewise, it is essential to design and build a strategic plan for the promotion of values, attitudes and ethical principles as a short or medium-term plan of action to educate the general population, particularly vulnerable groups. This promotion of values could be the cornerstone of a comprehensive and effective educational plan running alongside an established program, to avoid losing resources, that is, unnecessary expenses in a budget focused on an educational campaign to prevent abuse. If the necessary education is not provided, the risk of standing outside of an ethical context is high; a context where moral values are reduced to the memorization of concepts that are not practiced or to a merely ethical positivism where our random emotions lead our actions. An actual knowledge has to be pursued for the implementation of principles with intelligence, specificity and consideration.

### Summary

There is no current evidence to support the contention that fundamental ethical principles are applied in many countries that carry out clinical investigations, particularly in Latin America and regarding aspects related to safeguarding the rights of individuals and the privacy of confidential information such as data proceeding from the human genome. So long as concrete measures are not taken, vulnerable groups will continue to be the most affected populations. Abuse-related factors are predictable and can be prevented by working hard and collectively committing to the solution. In the advent of the genomics era, it is urgent to start closely monitoring compliance with fundamental ethical principles for research to prevent having massive leaks of information along with mistreatment, harm and violation of fundamental ethical principles, particularly in vulnerable groups.

## Abbreviations

IC: Informed Consent.

## Competing interests

The authors declare that they have no competing interests.

## Authors’ contributions

BC Design and writing of the manuscript, performance of data research. GG Coordination and help in drafting the manuscript. All authors read and approved the final manuscript.

## Pre-publication history

The pre-publication history for this paper can be accessed here:

http://www.biomedcentral.com/1472-6939/13/35/prepub
